# Updating the AIHTS Trapping Standards to Improve Animal Welfare and Capture Efficiency and Selectivity

**DOI:** 10.3390/ani10081262

**Published:** 2020-07-24

**Authors:** Gilbert Proulx, Marc Cattet, Thomas L. Serfass, Sandra E. Baker

**Affiliations:** 1 Alpha Wildlife Research & Management Ltd., 229 Lilac Terrace, Sherwood Park, AB T8H 1W3, Canada; 2 RGL Recovery Wildlife Health & Veterinary Services, 415 Mount Allison Crescent, Saskatoon, SK S7H 4A6, Canada; rgloperations.mcattet@gmail.com; 3 Department of Biology and Natural Resources, Frostburg State University, Frostburg, MD 21532, USA; TSerfass@frostburg.edu; 4 Wildlife Conservation Research Unit, Department of Zoology, The Recanati-Kaplan Centre, University of Oxford, Tubney House, Abingdon Road, Tubney, Abingdon OX13 5QL, UK; sandra.baker@zoo.ox.ac.uk

**Keywords:** AIHTS, animal welfare, capture efficiency, capture selectivity, humaneness, international trapping standards, International Organization for Standardization (ISO), mammals, trapping, wildlife management

## Abstract

**Simple Summary:**

The Agreement on International Humane Trapping Standards (AIHTS) has been the benchmark for humane restraining and killing traps used for the capture of a wide range of mammalian species for more than 20 years. Nonetheless, over this time, wildlife biologists, managers, and conservation groups have voiced a growing list of concerns about the ineffectiveness of AIHTS standards and test procedures in ensuring animal welfare. In this review, we first summarize and compare the AIHTS standards with two other contemporary standards, those developed by the International Organization for Standardization (ISO) in the late 90s and a Canadian trap research and development program in operation from 1985–1993. We then evaluate the AIHTS standards against seven hypotheses that reflect many of the concerns raised by the scientific community. Our evaluation shows conclusively that the AIHTS standards do not reflect state-of-the-art trapping technology and that continued maintenance of these outdated standards perpetuates animal pain and suffering. Lastly, we propose a series of measures to fund trap testing and implement new standards to improve animal welfare, and trap efficiency and selectivity.

**Abstract:**

In 1999, after pressure from the European Union, an Agreement on International Humane Trapping Standards (AIHTS) that would result in the banning of the steel-jawed leghold traps in the European Community, Canada, and Russia was signed. The United States implemented these standards through an Agreed Minute with the European Community. Over the last two decades, scientists have criticized the AIHTS for (1) omitting species that are commonly trapped; (2) threshold levels of trap acceptance that are not representative of state-of-the-art trap technology; (3) excluding popular traps which are commonly used by trappers although they are known to cause prolonged pain and stress to captured animals; (4) inadequate coverage of capture efficiency and species selectivity (i.e., number of captures of target and non-target species) performance. Concerns about the ability of standards and test procedures to ensure animal welfare, and about the implementation of standards, have also been voiced by wildlife biologists, managers, and conservation groups. In this review, we present a synopsis of current trapping standards and test procedures, and we compare the standards to a then contemporary 1985–1993 Canadian trap research and development program. On the basis of the above-noted concerns about AIHTS, and our experience as wildlife professionals involved in the capture of mammals, we formulated the following hypotheses: (1) the list of mammal species included in the AIHTS is incomplete; (2) the AIHTS have relatively low animal welfare performance thresholds of killing trap acceptance and do not reflect state-of-the-art trapping technology; (3) the AIHTS animal welfare indicators and injuries for restraining traps are insufficient; (4) the AIHTS testing procedures are neither thorough nor transparent; (5) the AIHTS protocols for the use of certified traps are inadequate; (6) the AIHTS procedures for the handling and dispatching of animals are nonexistent; (7) the AIHTS criteria to assess trap capture efficiency and species selectivity are inappropriate. We conclude that the AIHTS do not reflect state-of-the-art trapping technology, and assessment protocols need to be updated to include trap components and sets, animal handling and dispatching, and trap visit intervals. The list of traps and species included in the standards should be updated. Finally, the concepts of capture efficiency and trap selectivity should be developed and included in the standards. Based on our review, it is clear that mammal trapping standards need to be revisited to implement state-of-the-art trapping technology and improve capture efficiency and species selectivity. We believe that a committee of international professionals consisting of wildlife biologists and veterinarians with extensive experience in the capture of mammals and animal welfare could produce new standards within 1–2 years. We propose a series of measures to fund trap testing and implement new standards.

## 1. Introduction

In 1987, the International Organization for Standardization (ISO), through Technical Committee 191, began to develop humane mammal trapping standards [1]. The objective of the committee was to recommend scientifically measurable species-specific animal welfare (humane) thresholds that reflected state-of-the-art (i.e., the latest and most sophisticated or advanced stage of a technology) trapping systems internationally [2].

In 1991, because no international humane trapping standards were yet available, the Council of the European Union (EU–political and economic union formerly established in 1993 from the incorporation of the European Communities; it encompasses 27 member states, but the United Kingdom left the European Union in January 2020) adopted the “Leghold Trap” Regulation 3254/91. This regulation prohibited (a) the use of leghold traps in the European community and (b) the introduction into the European community of pelts and manufactured goods from countries that capture animals by using leghold traps or trapping methods that do not meet international humane trapping standards [3], which, at the time, still needed to be developed and approved.

In 1995, under pressure from the EU, negotiations began toward an Agreement on International Humane Trapping Standards (AIHTS) that would result in the banning of steel-jawed leghold traps in the territories of signatory countries [2]. This agreement was signed by the European Community, Canada, and Russia in 1997 [3]. The United States of America implemented humane trapping standards through an Agreed Minute with the European Community, which virtually replicated the AIHTS text [4,5]. According to the AIHTS and the Agreed Minute, restraining and killing traps used for the capture of members of certain mammalian species, traded among the parties for their fur, should be certified in accordance with a set of standards contained in the AIHTS [3,4].

The AIHTS is a binding agreement that has a direct impact on fur trading between the signatory parties [3]. In these countries, the AIHTS identifies certified traps to address animal welfare concerns associated with trapping. Although the AIHTS adapted some of the ISO testing procedures [6,7] to develop their own standards, the ISO standards have no legal values or enforcement capabilities. The use of ISO standards is voluntary and has no impact on the trade of goods or the legitimacy of traps used to capture mammals.

The AIHTS standards are now more than 20 years old. Over this time, scientists have criticized these AIHTS for (1) omitting species that are commonly trapped [8]; (2) threshold levels of acceptance that are not representative of state-of-the-art trap technology [9,10]; (3) excluding commonly used traps which are known to cause prolonged pain and stress to captured animals [11,12]; (4) inadequate guidelines to assess capture efficiency and species selectivity (i.e., number of captures of target and non-target species) performance [13]. Concerns have been voiced by wildlife biologists, managers, and conservation groups about the ineffectiveness of standards and test procedures in ensuring animal welfare [14] and in implementing standards [12,15,16]. Refinement of the international trapping standards is required to ensure that they generate desirable outcomes for animal welfare and do not preclude the development of improved methods [17].

Considering the growing concerns of the public and the scientific community about the welfare of wildlife [18,19,20], we believe that the time has come to review trapping standards, which are now outdated from both a scientific [9,10] and societal [21,22] point of view. On the basis of the above-noted concerns about AIHTS, and our experience as wildlife professionals involved in the capture of mammals, we formulated the following hypotheses: (1) the list of mammal species included in the AIHTS is incomplete; (2) the AIHTS have relatively low animal welfare performance thresholds of killing trap acceptance and do not reflect state-of-the-art trapping technology; (3) the AIHTS animal welfare indicators and injuries for restraining traps are insufficient; (4) the AIHTS testing procedures are neither thorough nor transparent; (5) the AIHTS protocols for the use of certified traps are inadequate; (6) the AIHTS procedures for the handling and dispatching of animals are nonexistent; (7) the AIHTS criteria to assess trap capture efficiency and species selectivity are inappropriate.

## 2. A Synopsis of Trapping Standards

We summarized the ISO standards [6,7] and the AIHTS [3] in Table 1 in order to provide the context for our subsequent review of issues and concerns. In order to understand our discussion of trap testing protocols included in standards, we briefly describe testing procedures below [10].

Whereas the AIHTS refer to “humane” standards, we believe this term to be inappropriate because, as wildlife professionals, we should be constantly striving for improvement [8,10,17,23]. Although frequently used in the scientific literature, this term may imply that humaneness is binary [24] and that, once a trap has been certified as “humane”, there is no need for further improvement. In this paper, unless the term has been cited in referred standards, we replace “humane standards” with “standards”.

### 2.1. Test Procedures

Mechanical evaluations: restraining and killing traps are tested in a laboratory to determine impact momentum and clamping forces. Impact momentum is the product of the velocity of a striking bar multiplied by its equivalent mass. The clamping force is the steady-state force exerted on an animal by the jaws of the trap after the striking force has been delivered. Mechanical evaluations of traps are important because they identify the maximum energy levels that may cause serious injuries in animals captured in restraining traps (e.g., foothold traps) or energy levels associated with time to irreversible unconsciousness (TIU) for animals struck in vital regions by killing traps [10,25]. Trap clamping force and impact momentum are widely accepted proxies of trap welfare performance among spring traps [6,10,25]. Past studies with killing traps showed that impact momentum and clamping force thresholds vary between strike locations within species and that neither force is directly related to target species bodyweight [26,27,28]. Mechanical forces cannot therefore be used to predict TIU between species based on their body weight. However, data on impact momentum and clamping force produced by a trap and the TIU for a certain species killed in that trap may be used in the screening of other traps for the same species, provided that the strike location is the same for both types of traps [25,26]. Mechanical evaluations allow one to perform quality control checks on mechanical traps [26] and assess how modifications of trap designs optimize the potential of a trap to restrain or kill an animal.

Compound tests with restraining traps: free-ranging animals in fenced compounds (semi-natural environments) are captured and kept in traps for ≤24h and are monitored for injuries and behavioural changes. Captured animals with severe injuries (e.g., fracture of bones or teeth, joint luxation, severance of tendons or ligaments, internal haemorrhages, corneal laceration, amputation, etc.) and signs of pain and suffering are euthanized immediately. Animals without apparent severe injuries are kept captive for the duration of the tests, euthanized, and necropsied. An assessment of the trap may be based on a cumulative scoring system, where points are assigned to the captured limb and body according to the severity of injuries [29].

Approach tests: tests conducted in compounds where the animals are allowed to approach traps wired in the set position so that the traps can be triggered but cannot close completely and injure the animals [30]. Approach tests are used to assess the ability of traps to strike animals in vital regions, e.g., the head and neck regions [31]. Traps that fail the approach tests should not be allowed to be tested further until they have been modified to strike animals properly.

Kill tests with anaesthetized animals: tests assessing the potential of traps to render anaesthetized animals irreversibly unconscious within a pre-determined time period. The muscles of anaesthetized animals are more relaxed than those of non-anaesthetized animals and offer less resistance to the striking bars than conscious animals that are fighting the trap [30,32]. Traps that successfully pass kill tests with anaesthetized animals may or may not pass killing tests in compounds. However, the probability of traps that failed kill tests with anaesthetized animals passing killing tests in compounds is null [30]. This was further confirmed through a statistical comparison of TIUs in kill tests with and without anaesthetized animals [33]. Thus, traps that fail kill tests with anaesthetized animals are not allowed to be further tested because they could not render non-anaesthetized animals unconscious quickly.

Kill tests in compounds: tests to assess the potential of traps to render mobile animals irreversibly unconscious within a pre-determined time period. All tests are recorded with video cameras and are monitored from afar. Upon firing of the trap, researchers run to the compound to monitor the state of consciousness (using the corneal and palpebral reflexes) of the trapped animals [30]. Animals are necropsied to determine pathological modifications associated with specific TIUs and trap strike locations [34].

Field tests (restraining and killing traps): test traps are evaluated, either alone (comparing capture efficiency to data reported for other traps) or in a comparison with commonly used traps [35], to ascertain compound test findings. One objective of field tests is to compare capture efficiency and species selectivity between experimental and commonly used traps. It is noteworthy to mention that traps which are commonly used by trappers may not meet animal welfare standards [36]. Animals are necropsied to compare trap strike locations and pathological modifications with those of animals killed in compound tests [36].

### 2.2. Comparisons

Since some of the ISO test procedures were adapted for the development of the AIHTS (Table 1), there are similarities between these standards (Table 1). The AIHTS are an improvement upon the ISO standards because they specify a minimum number of specimens and tests in compounds and a minimum number of successful tests to accept a trap from an animal welfare perspective (Table 1). However, the AIHTS fail to specify a minimum of successful outcomes in approach tests to ensure that traps have the potential of striking animals in vital regions. Although the AIHTS do not include either mechanical evaluations or kill tests with anaesthetized animals to eliminate killing traps with low killing potential, the standards indicate that ISO procedures should be used as appropriate. However, as we previously indicated, the ISO standards have no legal values.

In order to assess whether the AIHTS reflect state-of-the-art-trapping technology, we compared these standards to the testing procedures and trap acceptance criteria of a Canadian trap research and development program that was conducted in parallel with the development of the ISO and AIHTS standards, from 1985 to 1993 (Table 1). The ISO committee members, many of whom were subsequently involved in the development of the AIHTS, were familiar with the then contemporary Canadian research program and its achievements [37].

## 3. Hypothesis 1: The List of Mammal Species Included in the AIHTS Is Incomplete

Since the AIHTS was developed in response to cruelty associated with the use of steel-jawed leghold traps in fur trapping [2], standards were understandingly developed for furbearers captured for the pelt market [3]. The AIHTS identified 19 furbearer species, ranging in size from stoat (*Mustela erminea*) to grey wolf (*Canis lupus*) (Table 1). However, the standards are about animal (mammal) traps and, therefore, the list of species covered by the AIHTS is incomplete and does not encompass the majority of species being trapped in Canada, USA, Russia, or the EU as either furbearers, pests, research animals, or for conservation purposes.

### 3.1. Furbearers

Many more species harvested for their fur should be added to the AIHTS’ list. Among mustelids, American mink (*Neovison vison*), which are currently excluded from the list, are among the most numerous and valuable furbearers captured in North America [38,39]. The fact that most mink and fox pelts come from captive fur stock [16] does not justify omitting these species from the AIHTS list. Additionally, although thousands of least (*Mustela nivalis*) and long-tailed (*Mustela frenata*) weasels, wolverines (*Gulo gulo*), striped skunks (*Mephitis mephitis)*, red foxes (*Vulpes vulpes*), Arctic foxes (*Vulpes lagopus*), red squirrels (*Tamiasciurus hudsonicus*), Eurasian red squirrels (*Sciurus vulgaris*), and coypus (*Myocastor coypus*) are legally trapped every year in Canada, the USA, and Scandinavian countries [38,39,40], they are likewise overlooked by the AIHTS [3] for unknown reasons.

Since the impact momentum and clamping force necessary to cause irreversible consciousness within a certain time frame vary among species and between strike locations within species [26,27,41], one cannot claim that traps developed for furbearers of similar size may be used for species not listed in the AIHTS. For example, traps developed for American martens (*Martes americana*) cannot be assumed acceptable for trapping American mink, which have a greater cervical musculature and stronger bones than martens [42]. Traps developed for small and medium-sized mammals cannot be used for the relatively smaller weasels, because they may strike animals in non-lethal locations and cause distress and painful deaths [43]. Additionally, a trap that may be suitable for trapping fishers (*Pekania pennanti*) [44] cannot be presumed to be adequate for the larger and stronger wolverine, as strike locations will differ between species of different size and behaviour.

Many killing traps have been developed and manufactured for furbearers that are not included in the AIHTS. These traps were developed and demonstrated to meet more stringent time thresholds to irreversible unconsciousness than the AIHTS [42,45,46,47,48,49]. Tests in compounds and on working traplines have shown that the C120 Magnum with pan trigger could render mink irreversibly unconscious in <3 min [42,45], and the Sauvageau 2001-8 rotating-jaw trap could render Arctic fox irreversibly unconscious in <3 min [46,48]. Finally, tests in compounds [47] and in the field [49] have shown that the Kania trap could render American red squirrels irreversibly unconscious in <2 min and was a valuable alternative to ineffective traps and snares. All these furbearers should be part of the AIHTS list of species.

### 3.2. Rodents

Many millions of brown rats (*Rattus norvegicus*) and house mice (*Mus musculus*) in urban areas [50], and thousands of fossorial rodents in agricultural fields [51,52], are trapped globally every year. We believe that these species should be listed in the AIHTS, although, of course, they are not furbearers and so are currently excluded.

### 3.3. Other Non-Furbearing Species

Other mammals not on the AIHTS furbearers list are also trapped in high numbers. For example, although traps for rabbits, grey squirrels (*Sciurus carolinensis*), the non-native American mink, and most other mammals trapped in the UK are regulated to AIHTS standards, they are not included in the AIHTS. Moreover, in Canada, new trapping devices for species not included in the AIHTS have been independently developed, tested, and recognized to exceed AIHTS welfare standards, such as the PG Trap for the northern pocket gopher (*Thomomys talpoides*) [53]. We support Talling and Inglis’ [8] assertion that all mammal traps should be subject to regulation and, as discussed above, with appropriate TIU thresholds.

### 3.4. Animals Used in Research and Conservation Programs

Millions of mammals are also being trapped yearly by researchers for various conservation, demographic, physiological, and behavioural studies [54,55]. Although such studies may be subject to research and publication guidelines and Animal Care and Use Committees [19,56,57], data on traps being used to sample populations have not necessarily been investigated from an animal welfare and selectivity point of view.

Ungulates are also trapped for research, relocation, or population control, but assessment of these traps [58,59] and trapping standards are lacking in the AIHTS.

## 4. Hypothesis 2: The AIHTS Have Relatively Low Animal Welfare Performance Thresholds of Acceptance of Traps and Do Not Reflect State-of-the-Art Trapping Technology

### 4.1. Thresholds of Acceptance

In order to be acceptable, restraining and killing traps must meet the highest possible animal welfare standards, and trap standards should be raised as developments in trapping technology allow. Traps must be subjected to thorough assessment programs with appropriate acceptance threshold levels. If such levels are so low that any trap available on the market can be certified, trap manufacturers will not be encouraged to improve trap welfare standards, and concerns about animal welfare in trapping will be perpetuated.

In the following, we used the 1985–1993 Canadian research and development program for comparison purposes because this program produced a significant number of restraining and killing traps for use with American martens, American mink, fishers, northern raccoons (*Procyon lotor*), Arctic foxes, Canada lynx (*Lynx canadensis*), and red squirrels [60] that were then, and still are today, representative of state-of-the-art technology. The Canadian research program implemented stringent acceptation criteria [10] indicating that restraining traps should, with 95% confidence, hold ≥70% of animals for ≤24 h, with ≤50 points scored for physical injury, i.e., without serious or severe injuries [9,29,60]; killing traps should, with 95% confidence, render ≥70% of captured animals irreversibly unconscious in ≤3 min [10,60,61] (Table 1).

In accordance with Russel and Bursch’s “3Rs” principle (replacement, reduction, refinement) [62], the Canadian researchers aimed to minimize the number of animals used in testing by employing the normal approximation to the binomial distribution (one-tailed testing) to estimate the real potential of traps [34]. In compound tests, when a trap kills nine out of nine animals according to specific criteria, the success rate is 100%. However, in the real world, with a population of hundreds or thousands of animals, it is inconceivable to suggest that the tested trap model would successfully kill 100% of the animals according to the specified criteria. With the normal approximation to the binomial distribution, however, researchers could predict the expected performance of the tested traps in a large population of captured animals. Canadian researchers used the following equation:

$$P(X)=\frac{n!}{X!\left(n-X\right)!}\;p^{x}q^{n-x}$$

where *n* is the number of independent tests. Each test may result in one of two outcomes, “success” or “failure”, with the probabilities *p* and *q*, respectively. In a compound killing experiment with one trap model and nine animals, if nine tests are successful (i.e., nine animals lost consciousness within the prescribed time limit), and therefore failure is zero, the probability of the trap being successful in a large population of animals is estimated at 71% (one-tailed test) [63,64,65]. The same conclusion is reached with 13 successes out of 14 tests (i.e., one failure), 18 out of 20 (two failures), or 21 out of 24 (three failures), etc. (Table 1). Thus, if a trap model successfully kills nine out of nine animals in compound tests, it can be expected, at a 95% confidence level, to kill ≥70% animals of a target species captured on traplines [34,45] (Table 1). In the Canadian research program, traps that met this acceptance criterion were allowed to proceed to field tests. Field tests on traplines resulted in a large number of target species captures, and strike locations and pathological evidence were used to confirm conclusions drawn from compound kill test threshold levels. For example, killing traps for American martens, American mink, and Arctic foxes that had met the acceptance threshold levels in compound tests successfully killed 99%, 97%, and 100% of the animals of each species, respectively, on traplines [36,45,46].

According to the AIHTS, successful restraining traps must capture at least 16/20 animals without serious injuries, thus suggesting that traps will be humane in at least 80% of captures [3]. However, on the basis of the normal approximation to the binomial distribution, the real potential of these AIHTS-certified traps on traplines is only 57%. Likewise, according to the AIHTS, successful killing traps must render unconscious 10/12 animals within a pre-determined time period, thus suggesting that traps will be humane in 80% of captures [3]. However, the real potential of the traps would only be 49%, i.e., they may fail in nearly 50% of captures. In the 1980s, the performance threshold used by the Canadian research team was considered to be the “gold standard” [60]. The AIHTS thresholds of acceptance are markedly lower than this 35-year-old “gold standard”, and, therefore, the AIHTS do not reflect state-of-the-art trapping technology. While amendments to the agreement may be proposed by the AIHTS Committee, or any signatory country, at any time [3], performance thresholds have not been updated since the signing of the agreement in 1997.

While a minimum performance of 70% is superior to the actual performance achieved by the AIHTS standards, we still believe that this threshold performance level is inadequate. For example, with a minimum performance level of 70%, 30,000 out of 100,000 captured animals could suffer long and painful deaths or experience restraining conditions that are unacceptable in real-life trapping situations. With the current AIHTS, however, the situation is even worse, i.e., 51,000 out of 100,000 captured animals could suffer long and painful deaths in killing traps, and 43,000 restrained animals may suffer unacceptable welfare conditions in restraining traps. Compared to the standards used by Canadian researchers [10], 21,000 and 13,000 (21% and 13%) more animals, respectively, would be subjected to poor welfare conditions when being killed or restrained according to the AIHTS.

### 4.2. Times of Irreversible Loss of Consciousness (TIUs)

According to the AIHTS, the use of certified traps should result in TIUs of 45 s in stoat and similar small species, 120 s in martens (*Martes* spp.), and 300 s in other furbearer species. However, Canadian researchers [10,60] developed traps for American red squirrels with a TIU of 25 s only [47], and of 78 and 81 s for larger species [44,66]. With new technology and materials developed in the last few decades, TIUs could potentially be reduced to less than 60 s for many species.

Several rat and mouse traps (e.g., some Fenn, BMI Magnum, DOC traps, Nooski, and Good nature A24 traps) are regulated in the UK [67]. However, these traps are only expected to meet a threshold for TIUs of 300 s, although much shorter TIUs can be achieved with small animals. For example, break-back traps for rats and mice are specifically exempt from regulation in the UK and, as far as we understand, generally unregulated globally [68]. Voluntary trap approval standards used for rats and mice in Germany, such as those of the German Environment Agency and the Blue Angel (Blauer Engel) ecolabel, have the following categories: category A if 80% of test animals lose consciousness in 30 s and 90% in 60 s; category B if 80% of test animals are unconscious in 60 s and 90% in 180 s [69]. The Gorilla mouse trap has been certified according to this standard [70].

Clearly, much lower TIUs are achievable, at least for some smaller species, than those set out in the AIHTS, and we believe that approval thresholds should be overhauled accordingly. In the widespread absence of regulation for break-back traps, many trapping devices are used by the public without any information on the ability of traps to strike animals in suitable locations or quickly render them unconscious [20]. Baker et al. [20] investigated the potential welfare performance of a wide selection of unregulated break-back traps for rats and mice available in the UK by measuring their mechanical performance. They demonstrated wide variation in mechanical performance among traps for each of the species, which implies wide variation in welfare performance. Baker and co-authors subsequently proposed a voluntary trap approval scheme for unregulated break-back traps in the UK [71,72].

An actual performance level >80% may not be unrealistic since this has been achieved in previous trap development programs [60,73]. Traps and lower TIUs that resulted from the Canadian research program demonstrate that the AIHTS are not representative of current state-of-the-art technology.

Since the AIHTS thresholds do not represent state-of-the-art technology, some traps have been accepted as best management practices by the USA. This is despite Canadian scientific evaluations demonstrating that these traps did not have the potential to render ≥70% of animals unconscious within 3 min (therefore not meeting the AIHTS threshold level of performance), and they would cause undue pain and suffering as a result [15]. Examples of such traps include the popular Conibear 120 rotating-jaw traps (see Appendix 5.1 of [10] for a description of different trap types) for American martens [74] and American mink [75], and the Conibear 220 rotating-jaw trap for northern raccoons [76], all of which have been found to cause lengthy periods of consciousness and distress in previous assessments [30,32,42].

### 4.3. Trap Exemptions

The AIHTS specify that the parties of the agreement may derogate from the agreed standards for the use of some traps, i.e., they may allow the use of non-certified traps if their decision does not undermine the objectives of the agreement, for any of the following purposes: (a) the interests of public health and safety; (b) protection of public and private property; (c) research, education, repopulation, reintroduction, breeding, or for the protection of fauna and flora; (d) using traditional wooden traps essential for preserving cultural heritage of indigenous communities. In Canada and the United States, non-certified trapping devices are currently being used in the field (G. Proulx, personal observations), although their use likely undermines the objectives of the agreement. In Canada, killing neck snares are an example of trap exemptions.

Although ISO [6,7] testing procedures were included in the AIHTS [3], the ISO draft standard for killing neck snares [77] was excluded from the AIHTS because these trapping devices are considered to be homemade (Article 7 of the AIHTS excluding traps made by individuals) [12]. However, many killing neck snares are manufactured commercially and sold on the open market [2], and they should therefore be considered commercial devices and included in the AIHTS. Killing neck snares are unable to quickly render canids unconscious [11,12]; indeed, the animals may struggle violently for hours before losing consciousness [2]. However, more than 100,000 red foxes, coyotes (*Canis latrans*), and wolves are trapped every year in Canada [78], mostly in killing neck snares [2]. Thousands more canids are snared in the United States [79]. Killing neck snares play an important role in the capture of thousands of furbearers, and they cause injuries as severe as those associated with the outlawed steel-jawed leghold traps: major subcutaneous soft tissue lacerations, severe internal organ damage, bone and tooth fractures, joint dislocations, and haemorrhages [11]. These trapping devices should have been included in the original AIHTS and should be included in future trapping standards to ensure that they meet the same scrutiny and TIU thresholds as other trapping devices.

Several other mammal trapping devices are not covered by the AIHTS. For example, underwater traps (killing devices or restraining traps used as killing devices in drowning sets) received little attention in the past, even though the adequacy of drowning as a killing method has been questioned [80,81]. Many types of traps are excluded because the fur of the target species is not traded, e.g., traps used with rats and mice, including break-back traps, glue boards, multi-capture mouse traps, and bamboo rat traps.

## 5. Hypothesis 3: The AIHTS Animal Welfare Indicators and Injuries Are Insufficient

Mason and Mendl pointed out that the assessment of animal welfare relies too often on subjective judgements [82]. Animal welfare indicators and injuries used in the assessment of traps must reflect the extent of scientific knowledge about the anatomy, physiology, and behaviour of sentient organisms. The AIHTS aimed to equip researchers with objective quantitative methods to assess welfare, such as an injury-scoring system for restraining traps [60] or TIUs for use with killing traps [3]. Nevertheless, recognizing that both nonlethal and lethal methods can affect welfare, Mellor and Reid developed the five domains model (originally based on the United Kingdom Farm Animal Welfare Council’s Five Freedoms) [83]. The model assists in identifying welfare impacts under the following domains: nutrition, environment, health, behaviour, and mental state [83,84]. These five areas could be integrated into the AIHTS to help better quantify the welfare impacts of trapping.

The assessment of restraining traps should not be limited to injuries incurred in a trap but should also include the long-term effects of trapping on animals that have been released by the trapper or researcher. For example, the No. 3 Victor Softcatch^TM^ padded foothold trap appeared to cause little or no injuries to Ruppell’s foxes (*Vulpes rueppellii*) when individual animal injury rates were considered [85]. However, subsequent cage trapping showed that foothold trapping could lower the survival rate of these foxes for a period of 6 months following their release [85]. Similarly, significant capture-related effects in ursids may go undetected at the time of capture, thus providing a false sense of the welfare of released animals [86]. Cattet et al. [87] reported on the incidental diagnosis of exertional (capture) myopathy in a grizzly bear (*Ursus arctos*) that died approximately 10 d after capture by leghold snare. The same researchers found that serum concentrations of aspartate aminotransferase (AST) and creatine kinase (CK), biochemical indicators of muscle injury, were above normal levels in a higher proportion of apparently uninjured grizzly bears and black bears (*U. americanus*) captured in leghold snares than those captured by helicopter darting or by barrel trap [86]. In addition, the rate of movements made by bears decreased below mean normal rate immediately after capture and then returned to normal only 3–6 weeks after release [86]. Researchers determined that grey wolves (*Canis lupus*) captured in foothold traps and cable restraint devices (modified neck snares with a stop to avoid asphyxiation) restricted their activity and movement patterns for 8–10 d following capture [88]. Such behavioural changes could have significant impacts on the reproduction performance and survival (e.g., by not securing their minimum daily food intake or maintaining the integrity of their territory) of animals that were recently captured.

In the case of restraining devices, physiological investigations (e.g., analysis of blood collected from captured animals in compound tests or when retrieving animals in field tests) may provide researchers with a better understanding of the general condition of trapped animals and be used to validate conclusions reached using injury-scoring systems [89]. It is possible that assessments based entirely on physical injuries may not be adequate to assess particular trapping devices [60]. In this regard, persistent changes in the hematology and serum biochemistry of grizzly bears have been shown to be directly related to the number of times a bear was captured by leghold snare and the time interval between sequential captures [90]. Physiological investigations (e.g., stress/glucocorticoid assessments) may assist in the selection of trapping devices for a specific conservation program [60]. The AIHTS recognized the need to study behavioural and physiological changes during and after trapping. Twenty years later, however, there are no acceptance criteria in place.

The AIHTS identify self-mutilation, excessive immobility, and unresponsiveness as behavioural indicators of poor welfare. This list of indicators is clearly inadequate to assess the welfare of restrained animals. Signs of distress may include vocalization, carnivores feeding on plant material because they are dehydrated or hungry, the posture of the animals from the beginning to the end of the capture period, and changes in the alertness of animals at sunrise or sunset or when other animals pass by, etc. [2]. Behavioural changes may be investigated during compound tests, or in the field, using recordings with video cameras [2,29].

Whereas a long-term effect of live trapping on released animals might be expected, the impact of killing devices on animals which escape must also be taken into consideration when assessing traps. For example, killing neck snares do not quickly render canids unconscious [12], and when neck-snared canids escape, they usually die from infection and/or starvation hours or days after escaping [2]. The probability of animals escaping from killing traps needs to be assessed as part of any approval process.

## 6. Hypothesis 4: The AIHTS Testing Procedures Are Neither Thorough Nor Transparent

All trapping devices that successfully passed mechanical evaluations, approach tests, and compound tests in the Canadian research program were also successful in field tests [36,45,46]. This stepwise approach (Table 1) ensured that traps would be properly tested and the number of animals used in tests would be minimized. Nevertheless, it was necessary to use some animals in compound tests to assess traps and determine pathological changes that are associated with specific strikes in vital regions. Such information cannot be obtained without the use of animals. For example, by comparing strike locations and lesions induced by the trap for compound- and field-caught American martens, it was possible to make strong inferences with respect to the welfare of field-trapped animals. In the absence of background data on traps from compound studies, it would have been nearly impossible to assess the probable TIUs using field-caught animals, except in cases where there were massive cranial fractures [36,91].

The AIHTS allow for the use of other scientifically proven methods as a substitute for the testing procedures reported in Table 1. Several trapping devices have been certified by the Canadian fur industry [92] on the basis of the AIHTS, using computer programs that allegedly replicate the interaction of a particular species with a computer-drawn trap, thus minimizing the number of live animals required for the assessment [93]. However, there are no peer-reviewed scientific publications on computer-certified traps. Predicting how animals approach traps varies among species and between trap models and can be accurate only after many hours of behavioural observations, as was documented during the development of traps for American martens [30] and fishers [94]. For example, Proulx and Barrett [44] showed that fishers can escape from a lateral head strike by twisting their head while pulling out of the trap at firing time. In addition, they found that only dorsoventral head strikes were successful if the trap could generate both a large impact momentum and a large clamping force. Without compound evaluations with live animals, computer analysts would not have been able to predict the fisher’s head twisting behaviour. In other words, a trapper could have used computer-certified trap models on a trapline and surmised that all head strikes were successful in quickly rendering fishers unconscious, whereas, in reality, some may have escaped with head injuries. Likewise, a series of compound tests showed that a narrow mousetrap with a striking bar powered by a coil spring could not render red squirrels irreversibly unconscious within a pre-determined time period. The manufacturer recommended setting the trap perpendicular to a pole on the expectation that a squirrel running up the pole would step on the trigger laid over the pole and would be struck by the striking bar. However, compound tests showed that the trap could not consistently strike red squirrels in vital regions because of the rapid movements made by the animals around the time that the trap was triggered. During a series of approach tests, the trap and its set were modified to bring the animals to a full stop at firing time when the trap was triggered. The trap, baited with a pine cone and equipped with side wings, then properly positioned squirrels for a head strike [47]. The pine cone must be small and placed between the tips of the trigger prongs in order to force the animals to keep their heads low and away from the coil spring, thus allowing for more powerful head strikes. The peculiarities of American red squirrel behaviour when encountering the trap could not have been identified with computer assessments. Tests involving live animals were necessary. Therefore, if computer-generated assessments must be used, information about the frequency of strikes in vital regions and the nature of pathological changes associated with strikes must be gathered in compound tests before allowing traps to be field tested.

All findings, but particularly those related to computer-generated assessments, must be published (preferably in peer-reviewed journals) to allow members of the scientific community to evaluate protocols and findings. This is also true for traps included in the best management practices of the USA, for which data on animal welfare, injuries, and TIUs are scarce or nonexistent (G. Proulx and T. Serfass, conclusion based on a search of documents). In the past, concerns regarding the reliability of research outcomes have led biomedical scientists to request that findings be reported in publications that include hypothesis generation, experimental design, control and execution, statistical analysis, discussion, and conclusion [95,96]. The same is required for research and assessments that lead to the certification of traps that may have significant impacts on the welfare of captured animals. For example, 25 peer-reviewed scientific papers, plus conference proceedings and technical reports, were published on the 1985–1993 findings of the Canadian research program [10,60]. In contrast, as far as we know, no peer-reviewed or technical reports were made available to the scientific community and the public on >190 traps certified by the Fur Institute of Canada [92] using the AIHTS. Trap assessment and development needs to be transparent, and no “certified” trap should be released without the publication of the findings.

## 7. Hypothesis 5: The AIHTS Protocols for the Use of Certified Traps Are Inadequate

**Figure 1:**
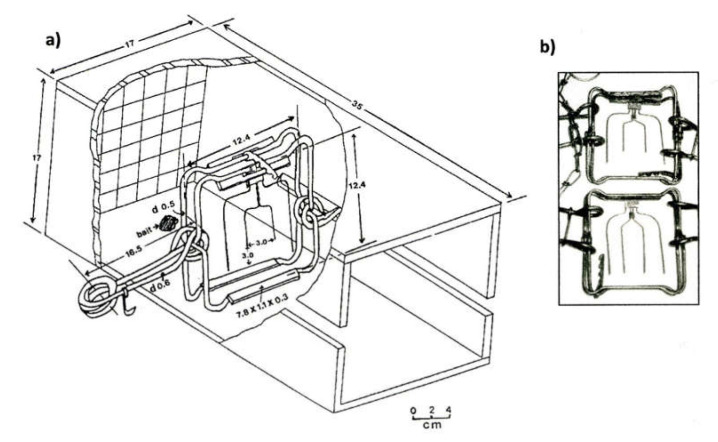
The ability of the C120 Magnum rotating-jaw trap to render American martens irreversibly unconscious in ≤3 min in compounds [34] and on traplines [36] was tested with a four-prong trigger and a specific cubby box (**a**) [60]. The trigger has two short centre prongs to properly position the animals in the traps and ensure a strike in vital regions [60]. When the original trigger was replaced with a four-long-prong trigger (**b**), the trap did not properly strike animals in the head [34] and was not as capture-efficient [98].

It is not sufficient to identify restraining traps that can hold animals with little injury or killing traps that can render animals unconscious quickly. Trap assessment must also include trap components and sets, as well as handling methods [10,97]. For example, when assessing the ability of the C120 Magnum rotating-jaw trap to render American martens irreversibly unconscious in ≤3 min, in enclosures and on traplines, researchers used a specific four-prong trigger and a cubby box set [34,36] (Figure 1). However, when equipping this trap with a different four-prong trigger, the trap did not properly strike animals in the head [34] and was not as capture-efficient [98]. Some trapping organizations now recognize that, in order to meet welfare objectives, traps must be equipped with specific triggers and set as in the assessment and development of the traps [99]. Manufacturers must recommend the triggers and sets that are specifically associated with the assessment of traps [10], and these should not be modified, as has often been observed, by trappers [100].

Trap components and sets should be considered as inherent elements of certified traps. They must be included in the certification of traps, and certified traps should not be sold, used, or equipped with components that differ from those used during the assessment. Indeed, when regulated spring traps are tested by the Animal and Plant Health Agency in the UK, they are tested in the set and housings, for which the manufacturer seeks approval. When approval is granted, it is dependent on specific conditions being met, e.g., regarding set or housing (S. Baker, personal notes).

## 8. Hypothesis 6: The AIHTS Procedures for the Handling and Dispatching of Animals Are Nonexistent

### 8.1. Animal Handling and Dispatching

The AIHTS does not address animal handling and dispatching. Many trappers promote professionalism in their trade and truly aim to minimize pain and suffering in captured animals [101,102]. Additionally, there are several examples of trappers whose actions to release non-target animals from their restraining traps exceed expectations [103]. However, too many trapper forums and pest control websites still suggest methods to kill trapped animals that are inappropriate, such as stunning with a stick and kneeling/stomping on the animal’s chest, strangulating with a loop at the end of a pole [104], or drowning [105]. Trappers also use small firearms, but they may shoot wolverines, wolves, and lynx in the chest to minimize damage to the pelt (and facilitate skinning) and protect the valuable skull [106]. Such methods diverge from methods that are recognized to minimize pain and distress.

There are many techniques for the appropriate handling and release of restrained animals [10,107]. When animals are still alive in killing traps, or in distress in restraining traps, handlers may euthanize animals <1 kg body weight with a blow to the head and larger ones with a gunshot to the head [10,108]. If animals can be sedated or anaesthetized, barbiturates and penetrating captive bolts may then be used [10,109]. Specific drugs and methods exist to kill animals [10,108], and some of them can be used in conjunction with sedatives or anaesthetics to minimize distress to captured animals while increasing handler safety. Appropriate animal handling and dispatching methods must be included in mammal trapping standards to ensure the welfare of animals and handlers. Without the proper handling of animals, there would be little point in improving traps.

### 8.2. Trap Visit Intervals

The AIHTS specify that, in field tests, traps should be checked daily. This means that the checking period could exceed 24 h if a trap is set or checked on the morning of one day and rechecked in the afternoon or evening of the following day [15]. Trap visitation rate is a fundamental factor in ensuring that certified traps and sets operate effectively. A recent review of legal trap-checking time periods showed that, in both Canada and the United States, checking times for restraining traps were usually once every 24 h [15]; in the real world, however, checking times often exceed such intervals (G. Proulx and T. Serfass, personal observations). The American Society of Mammalogists recommends that restraining traps for nocturnal species should be set before dusk and checked as soon as possible after dawn, while restraining traps for diurnal species should be set at dawn or early morning and checked every few hours in warm weather [56]. However, since some animals may injure themselves soon after capture [29], restraining traps should be visited at short time intervals and, ideally, as soon as possible following capture. This is particularly true for restraining snares, which are highly promoted in the USA, even though comprehensive field research on breakaway devices to protect non-target species is lacking [110]. In this regard, trap alarms can be used to alert handlers by mobile phone that a set trap has been triggered [111,112]. We realize that fur trappers’ views on trapping may differ from those of wildlife professionals [113,114]. However, from an animal welfare point of view, the end result of long trap visit intervals by researchers or fur trappers is the same, i.e., animals suffer. Therefore, the expectations of standards for trappers should aim to match those for scientific researchers.

Species vary in how well they cope with being held in restraining traps. For example, European moles (*Talpa europaea*) have a high metabolic rate and may die if left for long periods without food, and so it has been recommended that live mole traps should be checked at least every 4 h [115]. Restraining traps and killing traps should each be certified for a certain frequency of trap-checking, and checking time intervals may need to be species-specific, depending on the trap and its intended use.

There are no legal requirements to check killing traps and snares in most Canadian provinces and territories [15]. In the UK, there is no specified legal requirement to check any killing or restraining trap, apart from kill-traps for leporids (rabbits and hares), snares (used for foxes and rabbits), and Larsen traps (used for catching corvids under General License), which each need to be checked once every 24 h. An offence may be committed, however, under the UK Animal Welfare Act 2006, if an animal in a trap is caused “unnecessary suffering” [116]. In the United States, in nearly 35% of jurisdictions, legal checking times for killing traps and snares exceed 24 h. In approximately 55% of American states, legal checking time intervals for submersed killing devices exceed 36 h [15]. Acceptable welfare performance of traps should be tied to trap-checking time intervals, e.g., 12 h for live-traps and 24 h (but preferably 12 h) for killing traps [15]. Currently, the AIHTS criteria for the use of certified traps in the field are therefore incomplete.

## 9. Hypothesis 7: The AIHTS Criteria to Assess Trap Efficiency and Species Selectivity Are Inappropriate

### 9.1. Capture Efficiency

Although capture efficiency is regarded as the most important trap characteristic by trappers [35,117], the AIHTS does not address this aspect of trapping in detail. However, the ability of traps to capture target species, without the risk of escapes, is an important aspect of animal welfare. An injured animal escaping from a trap will suffer and possibly experience a long and painful death. The efficiency of a trap to hold captured animals should therefore be considered in the assessment of traps.

In 1999, however, ISO included protocols to assess the ability of traps to capture target animals in the field by recording the number of target animals caught by test traps and control traps (i.e., traps most popular among trappers, which may vary among regions). For example, because the experimental C120 Magnum rotating-jaw trap for American martens met the threshold level of acceptance in compound tests [34], it was tested on two traplines against control traps selected by the trappers [36]. Trappers selected all control traps and their sets. On the first trapline, control traps were factory-made Conibear 120, 126, and 160 rotating-jaw traps on a running (leaning) pole set. On the second trapline, control traps were the No. 3 coilspring and No. 4 longspring traps, placed in the entrance of a cubby box on a pole set. The number of C120 Magnum and control traps was the same on each trapline, and all traps were baited with beaver meat. For each target and non-target animal captured, a detailed set of measurements regarding trap placement on the animal, signs of struggle, oral or anal discharge, and pelt damage were recorded [36].

Many factors that affect trap efficiency, such as trap type, trap set, bait and lure, number of traps per unit area, and visitation rate, may be standardized in tests. Others are more difficult to standardize, such as trappers’ experience and trap use learning curve and environmental conditions [35], and these may bias the assessment of trap efficiency. Furthermore, the ISO [6,7] protocol does not account for the fact that the species assemblage and relative species abundance in test areas may vary among regions, and some abundant non-target species in a particular region may be more attracted to test traps than control traps, and vice versa, thus biasing the true assessment of capture efficiency. The results of a comparison of two trap types in one area cannot simply be extrapolated elsewhere.

In the past, some traps which were independently approved on welfare grounds in various research programs have been hastily and incorrectly rejected because of efficiency concerns (compare [118,119] and [120,121]). The ISO standards do not specify an acceptable percentage of efficiency [6,7]. However, the Canadian General Standards Board stipulated that the efficiency of a test trap must be at least 80% of the efficiency of a control trap [122]. Knowing that a control trap may vary in efficiency from one trapline to another, among years, and between trappers, and after considering all the above-listed factors that impact on capture success, this efficiency standard appears to be rather arbitrary [35]. It is also misleading because the acceptance level of capture efficiency may decrease over time. For example, an experimental trap T_1_ will be accepted if it meets 80% of the capture efficiency of a control trap T_2_. The newly accepted trap T_1_ could become a control trap in future assessments, and a new experimental trap T_3_ would have to meet 80% of the capture efficiency of T_1_, i.e., 64% of the capture efficiency of the original control trap T_2_. This compound decrease in capture efficiency could lead to the acceptance of traps that are not suitable to capture a particular species. We believe that field testing must be conducted at the regional level, ideally with known population densities for the target species [123] and with a series of trappers, in order to ensure that traps are successful at capturing target species without impacting on non-target species, particularly those at risk. This leads us to the subject of trap selectivity.

### 9.2. Species Selectivity

Although the AIHTS suggest that field testing of traps should include an assessment of trap selectivity (ability to capture members of the target species over members of non-target species), they provide no guidance on how this should be done [3]. However, when traps are not selective and capture individuals of non-target species, they may cause pain, distress, or death, and non-target animals may be handicapped for life after being captured in a trap set for another species. Use of unselective traps can also harm vulnerable populations of non-target species.

The ISO guidelines provide guidance on testing trap selectivity. The stated principle is to evaluate, in the field, the capability of a trap to capture target rather than non-target animals by recording the number of each that is captured by the test trap and by a control trap [6,7]. Virgós et al. [13] reviewed these guidelines and found them inadequate because (1) the ISO definition of selectivity does not account for the relative abundance of target and non-target species and does not therefore meaningfully reflect selectivity; (2) the guidelines’ methodology at best quantifies the relative selectivity of one trap against another, which is of limited use unless the control trap is known to have an acceptable level of absolute selectivity for the target species; (3) as with capture efficiency, information on relative trap selectivity cannot simply be extrapolated elsewhere, unless species assemblage and relative species abundances are consistent. The ISO definition of trap selectivity in effect provides only a simple capture proportion and therefore does not represent trap selectivity [13]. The impact of this is that authorities may, on the basis of ISO selectivity tests, inadvertently grant legal approval for the use of traps that are non-selective. This was the case in Spain, where the use of traditional snares that were allegedly selective for red foxes [124] accounted for the largest proportion of recorded mortality of the most endangered Iberian predator, the Iberian lynx (*Lynx pardinus*) [125,126]. In Canada, the use of killing neck snares aimed at killing various wild canids commonly leads to the deaths of many ungulates and carnivores, including species at risk, such as the grizzly bear and the woodland caribou (*Rangifer tarandus*) in Alberta, the wolverine in Quebec, and the lynx in Nova Scotia, [2,12]. Across Canada, domestic dogs (*Canis lupus familiaris*) are also found dead in neck snares set near private land [2]. In the UK, human runners have become entangled in restraining snares set for foxes [127].

Tested traps should be assessed with sets that are highly selective [44,123]. Trail sets, where a trap is laid across an animal’s path, are often used with restraining traps and killing neck snares or with small killing traps for species such as muskrats (*Ondatra zibethicus*). These sets are not selective and should not be considered in the development of acceptable traps.

## 10. Discussion

The development of the original international humane trapping standards (AIHTS) lasted nearly two decades, and discussions among countries and committees often focused on the maintenance of industrial activities such as furbearer trapping and the transformation of pelts into garments [37,128]. Furthermore, discussions were often re-directed by factions concerned only with animal rights and the abolishment of all trapping activities [128]. Conversely, some participants did not want to implement any restrictive standard and requested that standards be representative of trapping technology currently available on the market [37]. Therefore, the main purpose of these international standards, i.e., to minimize animal welfare impacts, was not always at the forefront of discussions. In addition, the implementation of state-of-the-art trap technology was continuously challenged by country representatives with diverse definitions of “humaneness” [37]. Not surprisingly, the resulting standards were even then incomplete, did not represent state-of-the-art technology, and were largely aimed at pleasing interest groups [37]. Nevertheless, they were a step towards the use of certified traps, and they improved communication and facilitated trade [3].

All the hypotheses that we formulated proved to be correct. Moreover, based on our review of the AIHTS, we believe that it is clear that mammal trapping standards need to be revisited to (1) include all trapped mammal species regardless of the reason for which they are captured; (2) implement state-of-the-art trapping technology, lower TIU thresholds for killing traps, and include all trapping devices and methods in current use; (3) expand on animal welfare indicators and injuries to detect poor animal welfare in animals captured in restraining traps; (4) improve trap testing procedures; (5) develop protocols on how to use certified traps and sets in the field; (6) develop protocols for the handling and dispatching of captured animals; (7) develop protocols to assess capture efficiency and species selectivity.

Over the last 20 years, countless datasets have been collected on the impact of trapping on animal welfare [10], trap selectivity and the impact of trapping on the persistence of animal populations [13,15], and the ethics of wildlife professionals and managers with respect to mammal trapping [2,9,10,72,97]. The development of better trapping standards should not require another 20 years due to a lack of definitions, a poor understanding of trapping research and development technology, or conceptual views about human–wildlife relationships. The maintenance of outdated standards and delays in implementing state-of-the-art technology simply perpetuate animal pain and suffering on an enormous scale.

Russel and Bursch’s “3Rs” principle should be applied to the use of animals in trap assessment [37]; this is already the case, for example, in the UK, where tests are abandoned as it becomes apparent that the threshold TIU for the approval of a particular trap cannot be met (S. Baker, personal notes). Recognized protocols for trap assessment, including referring to long-term physiological and behavioural datasets, exist and can be used to improve mammal trapping standards [10,84,129]. Mechanical evaluation must be used to assess the potential of traps and reduce the number of animals used in trap assessment [10]. If a trap developed and/or marketed by an inventor or a corporation that meets acceptance criteria is being copied (e.g., in terms of its dimensions and materials) by another company, the replicate could be evaluated mechanically to avoid using more animals in tests. Likewise, acceptable traps lose their power over time and should be replaced when their impact momentum and clamping forces fall below an acceptable level. The life expectancy of traps could be determined with a waveform analyzer [130], and traps that fail to meet the necessary energy levels could be refurbished with new springs or discarded.

Compound tests with animals are necessary to ensure that the majority of animals are properly captured and to avoid the possibility of hundreds, or even thousands, of animals suffering on traplines during field tests or subsequent use. Field tests are essential to further test conclusions reached in compound tests [10]. All trap tests and results should be published and made available for public scrutiny and scientific peer review.

Taking into consideration the concerns of environmental and conservation advocacy groups, trade organizations, and government socio-economic objectives, a committee of international professionals (i.e., wildlife biologists and veterinarians) [131] with extensive experience in the capture of mammals and animal welfare would be able to produce new standards within 1–2 years. However, the main focus must be on animal welfare and capture efficiency and selectivity. Trap testing with the new standards must be carried out by recognized authorities who are totally independent of trade organizations (e.g., pelt market, trapping organizations, inventors). However, the costs associated with the development of new standards, the development of traps meeting these standards, and the implementation of the standards in the field (see below) should be covered by trap manufacturers and retailers, fur buyers and retailers, government agencies, and “user-pay” levies, where trappers and pest controllers pay an extra fee when purchasing trapping licenses, traps, and guns [132].

Undue delays in the implementation of standards [16,50,133] must be eliminated, and the implementation of the standards must be enforced at all levels, e.g., trapping supply markets including e-commerce businesses, traplines, and fur markets. Only effective trapping systems (i.e., traps with their trigger and set) will be used in the field if trap markets, traplines, and fur buyers are closely monitored by recognized agencies. Trappers and researchers need to be trained in certified trapping methods by individuals who understand trapping standards and animal welfare; online video teaching would be a valuable option. Traps need to be individually identified (e.g., registration number, telephone number, etc.) and dated to determine if they need to be refurbished or discarded. All captures (target and non-target) must be reported to the relevant authorities, and field inspections must be conducted by independent agencies which report to the committee in charge of the new trapping standards and signed agreement. New improved standards need to be widely implemented in all signatory countries, and the rule of law should be properly enforced.

We hope that this review will encourage animal welfare and conservation experts to (1) recruit leaders in trapping research and development to establish a committee that will oversee the elaboration of state-of-the art international mammal trapping standards for the 21st century; (2) approach the AIHTS signatory countries to agree to a review of standards to improve animal welfare and trap efficiency and selectivity; (3) implement a time-efficient schedule to redraft standards, test traps according to new standards, refurbish or produce new traps, teach trappers and researchers about the new standards, and establish an implementation program to enforce the new standards.

## 11. Conclusions

Our review of the AIHTS showed that (1) the list of mammal species included in the AIHTS is incomplete; (2) the AIHTS have relatively low animal welfare performance thresholds of killing-trap acceptance, and do not reflect state-of-the-art trapping technology; (3) the AIHTS animal welfare indicators and injuries for restraining traps are insufficient; (4) the AIHTS testing procedures are neither thorough nor transparent; (5) the AIHTS protocols for the use of certified traps are inadequate; (6) the AIHTS procedures for the handling and dispatching of animals are nonexistent; (7) the AIHTS criteria to assess trap capture efficiency and species selectivity are inappropriate. The AIHTS trapping standards must be updated to improve animal welfare, and trap efficiency and selectivity.

## Figures and Tables

**Table 1:** Comparison of International Organization for Standardization (ISO) and Agreement on International Humane Trapping Standards (AIHTS) (excerpts from the original documents) to a Canadian research protocol conducted in parallel with the development of the standards.

Subject	Standards				1985-93 Canadian Research Program [10]	
	ISO [6,7]		AIHTS [3]			
	Restraining Traps	Killing Traps	Restraining Traps	Killing Traps	Restraining Traps	Killing Traps
Legal significance	None		Binding agreement—each party should take the necessary steps to ensure that the respective competent authorities (a) establish appropriate processes for certifying traps in accordance with the standards; (b) ensure that the trapping methods conducted in their respective territories are in accordance with the standards; (c) prohibit the use of traps that are not certified in accordance with the standards; (d) require manufacturers to identify certified traps and provide instructions for their appropriate setting, safe operation, and maintenance.		None	
Definition	Device used to capture and restrain a mammal. A restraining trap system encompasses equipment (trap and trigger) and set (site modifications, lures, and baits).	Device for use on land or underwater to kill a mammal. A killing trap system encompasses equipment (trap and trigger) and set (site modifications, lures, and baits).	Traps designed and set with the intention of not killing the trapped animal but restricting its movements to such an extent that a human can make direct contact with it.	Traps designed and set with the intention of killing a trapped animal of the target species.	As per ISO and AIHTS	
List of species	All mammal species		Coyote (*Canis latrans*) Wolf (*Canis lupus*) North American beaver (*Castor canadensis*) European beaver (*Castor fiber*) Bobcat (*Felis rufus*) North American otter (*Lontra canadensis*) European otter (*Lutra lutra*) Canada lynx (*Lynx canadensis*) European lynx (*Lynx lynx*) American marten (*Martes americana*) Fisher (*Pekania pennanti*) Sable (*Martes zibellina*) Pine marten (*Martes martes*) European badger (*Meles meles*) Ermine (*Mustela erminea*) Raccoon dog (*Nyctereutes procyonoides*) Muskrat (*Ondatra zibethicus*) Raccoon (*Procyon lotor*) North American badger (*Taxidea taxus*)		All mammal species Species for which acceptable traps have been developed by researchers according to performance levels presented below: Arctic fox (*Vulpes lagopus*) Red squirrel *(Tamiasciurus hudsonicus)* Northern pocket gopher (*Thomomys talpoides*) Canada lynx American marten Fisher American mink (*Neovison vison*) Raccoon	
Testing procedure	Field testing Pathological evaluation	Mechanical evaluation Kill tests with anaesthetized animals. Kill tests in compounds. Field tests. Pathological evaluations. Inspection and testing for user safety and traps.	Compound tests to evaluate behavioural, physiological, and biochemical parameters. Field tests (vs. a control trap) to assess selectivity and user safety. Pathological evaluations.	Approach tests to ensure a proper positioning of the animals in the traps. Kill tests in compounds to assess loss of consciousness. Field tests (vs. a control trap) to assess selectivity and user safety. Pathological evaluations.	Mechanical evaluation to compare impact momentum and clamping forces of different trap models. Compound tests to assess behavioural and physiological parameters. Field tests (vs. a control trap) to assess selectivity and user safety. Pathological evaluations.	Mechanical evaluation to assess the potential of traps.Approach tests to ensure a proper positioning of the animals in the traps. Kill tests with anaesthetized animals to assess loss of consciousness in non-reactive animals. Kill tests in compounds to assess loss of consciousness in conscious animals. Field tests to verify compound test findings. Pathological evaluations in compound and field tests. Inspection and testing for user safety and traps.
Test report	Humaneness: report on the position of each animal in the trap and evaluation of the condition of the captured animals according to a trauma scale, with scores proportional to the severity of the injuries. Field tests: number of captures of target and non-target species. User safety: field notes.	Humaneness: report on strike location and time to loss of corneal and palpebral reflexes and heartbeat. Field tests: number of captures of target and non-target species. Compound and field tests: pathological observations. User safety: field notes.	Humaneness Behavioural indicators of poor welfare: Self-mutilation Excessive immobility and unresponsiveness. Physical indicators of poor welfare according to occurrence of serious and severe injuries.	Humaneness The time of occurrence and insensibility produced by the killing technique based on the loss of corneal and palpebral reflexes or any other specifically proven suitable substitute parameter: 45 s—*Mustela erminea* 120 s—*Martes* spp. 300 s—all other species listed above.	Humaneness: report on the position of each animal in the trap and evaluation of the condition of the captured animals according to a trauma scale with scores proportional to the severity of the injuries; total injuries must amount to <50 points on the scale. Field tests: Number of captures of target and non-target species. User safety: field notes.	Humaneness The number of animals tested and the proportion that lost insensibility based on the loss of corneal and palpebral reflexes. 3 min—all species except large carnivores. 5 min—Red fox (*Vulpes Vulpes*)
Number of tests	Unspecified. The number of replicates in the tests shall be sufficient to determine if the differences are statistically significant at the level to be determined by the authority implementing the test. Comparison of selectivity (number of captured target animals divided by the total number of captured animals) with a control trap and user safety as specified by the authority implementing the standard.	Unspecified. Capability of a killing trap, as part of the killing trap system, to kill an animal within a time period and to meet the requirements related to mechanical properties, comparison of selectivity (number of captured target animals divided by the total number of captured animals) with a control trap model, and user safety as specified by the authority implementing the standard.	The number of specimens of the same target species from which the data are derived is at least 20.	The number of specimens of the same target species from which the data are derived is at least 12.	≥9 specimens for compound tests. >30 specimens for field tests where capture durations ≤24 h.	≥6 specimens for approach tests ≥9 specimens for compound kill tests. >30 specimens for field tests.
Minimum successful compound tests required to meet performance thresholds	None	None	At least 16 (80%) of 20 animals show none of the indicators listed above.	At least 10 (80%) of 12 animals are unconscious and insensible within the time limit and remain in this state until death.	9/9 (100%), or 13/14 (93%), or 21/24(88%), etc. (proportions based on the normal approximation to the binomial distribution).	
Predicted performance threshold at population level (95% confidence level) resulting from the number of successful tests—one-tailed binomial test	n/a	n/a	57%	49%	71%	

## References

[1] Barrett M.W., Proulx G., Jotham N. (1988). Wild fur industry under challenge–the Canadian response. Trans. North Am. Wildl. Nat. Resour. Conf..

[2] Proulx G. (2018). Intolerable Cruelty—The Truth behind Killing Neck Snares and Strychnine.

[3] ECGCGRF (European Community, Government of Canada, and Government of the Russian Federation) (1997). Agreement on international humane trapping standards. Off. J. Eur. Communities.

[4] (1998). International agreement in the form of an agreed minute between the European Community and the United States of America on humane trapping standards. Off. J. Eur. Communities.

[5] Harrop S.R. (1998). The agreements on international humane trapping standards—Background, critique and the texts. J. Int. Wildl. Law Policy.

[6] ISO 10990-4 (1999). Part 4: Methods for testing killing trap systems used on land and underwater. Animal (Mammal) Traps.

[7] ISO 10990-5 (1999). Part 5: Methods for testing restraining traps. Animal (Mammal) Traps.

[8] Talling J.C., Inglis I.R. (2009). Improvements to Trapping Standards. DG ENV. http://ec.europa.eu/environment/biodiversity/animal_welfare/hts/pdf/final_report.pdf.

[9] Powell R.A., Proulx G. (2003). Trapping and marking terrestrial mammals for research: Integrating ethics, standards, techniques, and common sense. Inst. Lab. Anim. Res. J..

[10] Proulx G., Cattet M.R.L., Powell R.A., Boitani L., Powell R.A. (2012). Humane and efficient capture and handling methods for carnivores. Carnivore Ecology and Conservation: A Handbook of Techniques.

[11] Proulx G., Rodtka D. (2017). Steel-jawed leghold traps and killing neck snares: Similar injuries command a change to agreement on international humane trapping standards. J. Appl. Anim. Welf. Sci..

[12] Proulx G., Rodtka D., Barrett M.W., Cattet M., Dekker D., Moffatt E., Powell R.A. (2015). Humaneness and selectivity of killing neck snares used to capture canids in Canada: A review. Can. Wildl. Biol. Manag..

[13] Virgós E., Lozano J., Cabezas-Díaz S., Macdonald D.W., Zalewski A., Atienza J.C., Proulx G., Ripple W.J., Rosalino L.M., Santos-Reis M. (2016). A poor international standard for trap selectivity threatens global carnivore and biodiversity conservation. Biodivers. Conserv..

[14] Iossa G., Soulsbury C.D., Harris S. (2007). Mammal trapping: A review of animal welfare standards of killing and restraining traps. Anim. Welf..

[15] Proulx G., Rodtka D. (2019). Killing traps and snares in North America: The need for stricter checking time periods. Animals.

[16] Game and Wildlife Conservation Trust (2020). Agreement on International Humane Trapping Standards (AIHTS). https://www.gwct.org.uk/advisory/faqs/aihts/.

[17] Hampton J.O., Hyndman T.H., Laurence M., Perry A.L., Adams P., Collins T. (2016). Animal welfare and the use of procedural documents: LImitations and refinement. Wildl. Res..

[18] Paquet P.C., Darimont C.T. (2010). Wildlife conservation and animal welfare: Two sides of the same coin?. Anim. Welf..

[19] Field K.A., Paquet P.C., Artelle K., Proulx G., Brook R.K., Darimont C.T. (2019). Publication reform to safeguard wildlife from researcher harm. PLoS Biol..

[20] Baker S.E., Ellwood S.A., Tagarielli V.L., Macdonald D.W. (2012). Mechanical performance of rat, mouse and mole spring traps, and possible implications for welfare performance. PLoS ONE.

[21] Jacobs M.H. (2007). Wildlife value orientations in the Netherlands. Hum. Dimens. Wildl..

[22] Manfredo M.J., Teel T.L., Dietsch A.M. (2016). Implications of human value shift and persistence for biodiversity conservation. Conserv. Biol..

[23] Proulx G., Barrett M.W. (1989). Animal welfare concerns and wildlife trapping: Ethics, standards and commitments. Trans. West. Sect. Wildl. Soc..

[24] Hampton J.O., Fisher P.M., Warburton B. (2020). Reconsidering humaneness. Conserv. Biol..

[25] Proulx G., Proulx G., Bryant H.N., Woodard P.M. (1997). Improved trapping standards for marten and fisher. Martes: Taxonomy, Ecology, Techniques and Management.

[26] Cook S.R., Proulx G. (1989). Mechanical evaluation and performance improvement of the rotating jaw Conibear 120 trap. ASTM J. Test. Eval..

[27] Zelin S., Jofriet J.C., Percival K., Abdinoor D.J. (1983). Evaluation of humane traps: Momentum thresholds for four furbearers. J. Wildl. Manag..

[28] Warburton B., Hall J.V. (1995). Impact momentum and clamping force thresholds for developing standards for possum kill traps. N. Z. J. Zool..

[29] Proulx G., Onderka D.K., Kolenosky A.J., Cole P.J., Drescher R.K., Badry M.J. (1993). Injuries and behavior of raccoons (*Procyon lotor*) captured in the Soft Catch^TM^ and the EGG^TM^ traps in simulated natural environments. J. Wildl. Dis..

[30] Proulx G., Cook S.R., Barrett M.W. (1989). Assessment and preliminary development of the rotating-jaw Conibear 120 trap to effectively kill marten (*Martes americana*). Can. J. Zool..

[31] Gilbert F.F., Chapman J.A., Pursley D. (1981). Assessment of Furbearer Response to Trapping Devices, Proceedings of the Worldwide Furbearer Conference, Frostburg, MD, USA, 3–11 August 1980.

[32] Proulx G., Drescher R.K. (1994). Assessment of rotating-jaw traps to humanely kill raccoons. J. Wildl. Dis..

[33] Hiltz M., Roy L.D. (2001). Use of anaesthetized animals to test humaneness of killing traps. Wildl. Soc. Bull..

[34] Proulx G., Barrett M.W., Cook S.R. (1989). The C120 Magnum: An effective quick-kill trap for marten. Wildl. Soc. Bull..

[35] Pawlina I., Proulx G., Proulx G. (1999). Factors affecting trap efficiency: A review. Mammal Trapping.

[36] Barrett M.W., Proulx G., Hobson D., Nelson D., Nolan J.W. (1989). Field evaluation of the C120 Magnum trap for marten. Wildl. Soc. Bull..

[37] Proulx G. (Alpha Wildlife Research & Management Ltd., Sherwood Park, Alberta, T8H 1W3, Canada). Unpublished notes gathered during his participation as a technical expert in ISO meetings in Brussels, Belgium, 1992.

[38] Obbard M.L., Jones J.G., Newman R., Booth A., Satterthwaite A.J., Linscombe G., Novak M., Baker J.A., Obbard M.E., Malloch B. (1987). Furbearer harvests in North America. Wild Furbearer Management and Conservation in North America.

[39] Proulx G., Griffiths H.I. (2000). The impact of human activities on North American mustelids. Mustelids in a Modern World: Management and Conservation Aspects of Small Carnivore: Human Interactions.

[40] Mørk T., Bohlin J., Fuglei E., Asbakk K., Tryland M. (2011). Rabies in the Arctic fox population, Svalbard, Norway. J. Wildl. Dis..

[41] Proulx G., Barrett M.W. A review of the 1985–88 humane trapping research program at the Alberta Environmental Centre, Vegreville, Alberta. Proceedings of the International Symposium on Trapping Wild Furbearers.

[42] Proulx G., Barrett M.W., Cook S.R. (1990). The C120 Magnum with pan trigger: A humane trap for mink (*Mustela vison*). J. Wildl. Dis..

[43] Drickamer L.C., Mikesic D.G. (1993). Differences in trapping and killing efficiency of Sherman, Victor and Museum Special traps for house mice. Am. Midl. Nat..

[44] Proulx G., Barrett M.W. (1993). Evaluation of the Bionic^®^ trap to quickly kill fisher (*Martes pennanti*) in simulated natural environments. J. Wildl. Dis..

[45] Proulx G., Barrett M.W. (1993). Field testing the C120 Magnum trap for mink. Wildl. Soc. Bull..

[46] Proulx G., Pawlina I.M., Onderka D.K., Badry M.J., Seidel K. (1994). Field evaluation of the No. 1-1/2 steel-jawed leghold and the Sauvageau 2001-8 traps to humanely capture Arctic fox. Wildl. Soc. Bull..

[47] Proulx G., Kolenosky A.J., Cole P.J. (1993). Assessment of the Kania^®^ trap to humanely kill red squirrels (*Tamiasciurus hudsonicus*) in enclosures. J. Wildl. Dis..

[48] Proulx G., Kolenosky A.J., Badry M.J., Cole P.J., Drescher R.K. (1993). Assessment of the Sauvageau 2001-8 trap to effectively kill Arctic fox. Wildl. Soc. Bull..

[49] Proulx G. (1996). Testing the ARC 90-16 Kania Trap for Red Squirrels on Traplines.

[50] Mason G., Littin K. (2003). The humaneness of rodent pest control. Anim. Welf..

[51] Proulx G. (1997). A northern pocket gopher (Thomomys talpoides) border control strategy: Promising approach. Crop Prot..

[52] Marsh R.E., Halverson W.S., Crabb A.C. (1994). Current (1994) ground squirrel control practices in California, Proceedings of the 16th Vertebrate Pest Conference, Santa Clara, CA, USA, 28 February, 1–3 March, 1994.

[53] Proulx G., Proulx G. (1999). Evaluation of the experimental PG trap to effectively kill northern pocket gophers. Mammal Trapping.

[54] Mengak M.T., Guynn D.C. (1987). Pitfalls and snap traps for sampling small mammals and herpetofauna. Am. Midl. Nat..

[55] Delehanty B., Boonstra R. (2009). Impact of live trapping on stress profiles of Richardson’s ground squirrel (*Spermophilus richardsonii*). Gen. Comp. Endocrinol..

[56] Sikes R.S., Gannon W.L., The Animal Care and Use Committee of the American Society of Mammalogists (2011). Guidelines of the American Society of Mammalogists for the use of wild mammals in research. J. Mammal..

[57] Proulx G. (2017). Animal welfare concerns in wildlife research and management. Can. Wildl. Biol. Manag..

[58] Beringer J., Hansen L.P., Sheriff S.L., Proulx G. (1999). Evaluation of two capture techniques for white-tailed deer. Mammal Trapping.

[59] Bergvall U.A., Jäderberg L., Kjellander P. (2017). The use of box-traps for wild roe deer: Behaviour, injuries and recaptures. Eur. J. Wildl. Res..

[60] Proulx G., Proulx G. (1999). Review of current mammal trap technology in North America. Mammal Trapping.

[61] Proulx G., Barrett M.W., Buskirk S.W., Harestad A.S., Raphael M.G., Powell R.A. (1994). Ethical considerations in the selection of traps to harvest martens and fishers. Martens, Sables, and Fishers: Biology and Conservation.

[62] Russell W.M.S., Burch R.L. (1959). The Principles of Humane Experimental Technique.

[63] Fleiss J.L. (1981). Statistical Methods for Rates and Proportions.

[64] Zar J.H. (1999). Biostatistical Analysis.

[65] Dulberg C. (Health Sciences, University of Ottawa, Ottawa, ON, Canada). Personal communication, 1992.

[66] Proulx G., Kolenosky A.J., Cole P.J., Drescher R.K. (1995). A humane killing trap for lynx (*Felis lynx*): The Conibear 330 with clamping bars. J. Wildl. Dis..

[67] The Spring Traps Approval (England) Order 2018. http://www.legislation.gov.

[68] Baker S.E., Sharp T.M. (2015). Welfare in commensal rodent trapping: One step forward, two steps back. Anim. Welf..

[69] Blue Angel—The German Ecolabel (2017). Non-Toxic Pest Control and Prevention DE-UZ 34 Basic Award Criteria, Edition January 2017, Version 2. https://produktinfo.blauer-engel.de/uploads/criteriafile/en/DE-UZ%20034-201701-en%20Criteria.pdf.

[70] Blue Angel—Rodent traps. https://www.blauer-engel.de/en/products/home-living/pest-control-biocide-free-indoor/rodent-traps.

[71] Baker S.E. (2017). A voluntary trap approval scheme to end trap welfare inequality in the UK. Anim. Welf..

[72] Baker S.E., Macdonald D.W., Ellwood S.A., Davies M.P., Pfeiffer C., Robinson W.H. (2017). Double Standards in Spring Trap Welfare: Ending Inequality for Rats (Rodentia: Muridae), Mice (Rodentia: Muridae) and Moles (Insectivora: Talpidae) in the United Kingdom, Proceedings of the Ninth International Conference on Urban Pests, Birmingham, UK, 9–12 July 2017.

[73] Pontu N., Warburton B. (2003). Evaluation of the Effectiveness of the Waddington Backcracker Trap for Killing Stoats.

[74] Association of Fish & Wildlife Agencies (AFWA] (2014). Best Management Practices for trapping American marten in the United States. Mimeograph. https://www.fishwildlife.org/application/files/.

[75] Association of Fish & Wildlife Agencies (AFWA) Undated. Best Management Practices for Trapping Mink in the United States. Mimeograph. https://www.fishwildlife.org/application/files/2015/2105/2663/MinkRV3.pdf.

[76] (2014). Association of Fish & Wildlife Agencies (AFWA). Best Management Practices for Trapping Raccoons in the United States. Mimeograph. https://www.dec.ny.gov/docs/wildlife_pdf/trapbmpsraccoon.pdf.

[77] ISO (1995). Part 4, Non-mechanically powered killing snares. Animal (Mammal Traps).

[78] Statistics Canada (2011). Fur Statistics.

[79] Fox C.H., Papouchis C.M. (2004). Cull of the Wild—A Contemporary Analysis of Wildlife Trapping in the United States.

[80] Andrews E.J., Bennett B.T., Clark J.D., Houpt K.A., Pascoe P.J., Robinson G.W., Boyce J.R. (1993). Report of the AVMA panel on euthanasia. J. Am. Vet. Med Assoc..

[81] Ludders J.W., Schmidt R.H., Dein F.J., Klein P.N. (1999). Drowning is not euthanasia. Wildl. Soc. Bull..

[82] Mason G., Mendl M. (1993). Why is there no simple way of measuring animal welfare?. Anim. Welf..

[83] Mellor D.J., Reid C.S.W., Baker R.M., Jenkin G., Mellor D.J. (1994). Concepts of animal well-being and predicting the impact of procedures on experimental animals. Improving the Well-Being of Animals in the Research Environment.

[84] Sharp T., Saunders G. (2011). A model for Assessing the Relative Humaneness of Pest Animal Control Methods.

[85] Seddon P.J., van Heezik Y., Maloney R.E., Proulx G. (1999). Short- and medium-term evaluations of foothold trap injuries in two species of fox in Saudi Arabia. Mammal Trapping.

[86] Cattet M., Boulanger J., Stenhouse G., Powell R.A., Reynolds-Hogland M.J. (2008). An evaluation of long-term capture effects in ursids: Implications for wildlife welfare and research. J. Mammal..

[87] Cattet M., Stenhouse G., Bollinger T. (2008). Exertional myopathy in a grizzly bear (*Ursus arctos*) captured by leghold snare. J. Wildl. Dis..

[88] Gese E.M., Terletzky P.A., Erb J.D., Fuller K.C., Grabarkewitz J.P., Hart J.P., Humpal C., Sampson B.A., Young J.K. (2019). Injury scores and spatial responses of wolves following capture: Cable restraints versus foothold traps. Wildl. Soc. Bull..

[89] Warburton B., Gregory N., Bunce M., Proulx G. (1999). Stress response of Australian brushtail possums captured in foothold and cage traps. Mammal Trapping.

[90] Cattet M., Wismer D., Stenhouse G. (2020). Wildlife Health: An Integral Component of Forest Management.

[91] Onderka D.K., Proulx G. (1999). Pathological examination as an aid for trap selection guidelines: Usefulness and limitations. Mammal Trapping.

[92] Fur Institute of Canada (FIC) (2019). Certified Traps—AIHTS Implementation in Canada. https://fur.ca/certified-traps/.

[93] Hiltz M., Roy L.D., Salmon L.P., Crabb A.C. (2000). Rating Killing Traps Against Humane Trapping Standards Using Computer Simulations. Proceedings of the 19th Vertebrate Pest Conference, San Diego, California.

[94] Proulx G., Barrett M.W. (1993). Evaluation of mechanically improved Conibear 220^TM^ traps to quickly kill fisher (*Martes pennanti*) in simulated natural environments. J. Wildl. Dis..

[95] Puhan M.A., Akl E.A., Bryant D., Xie F., Apalone G., Ter Riet G. (2012). Discussing study limitations in reports and biomedical studies—The need for more transparency. Health Qual. Life Outcomes.

[96] Jarvis M.F., Williams M. (2016). Irreproducibility in preclinical biomedical research: Perceptions, uncertainties, and knowledge gaps. Trends Pharm. Res..

[97] Serfass T.L., Wright L., Pearce K., Duplaix N., Butterworth A. (2017). Animal welfare issues pertaining to the trapping of otters for research, conservation, and fur. Marine Mammal Welfare.

[98] Naylor B.J., Novak M. (1994). Catch efficiency and selectivity of various traps and sets used for capturing American martens. Wildl. Soc. Bull..

[99] Fournier G., Canac-Marquis P. Best Trapping Practices. Fédération des trappeurs gestionnaires du Québec: Québec, QC, Canada, 2018. https://mffp.gouv.qc.ca/wp-content/uploads/best-trapping-pratices-juillet-2018.pdf.

[100] Utah Today’s Trapper Course American Marten Sets. https://www.hunter-ed.com/utah/studyGuide/American-Marten-Sets/221046_700121056/.

[101] Giroux A., Novak M., Baker J.A., Obbard M.E., Malloch B. (1987). The role of the trapper today. Wild Furbearer Management and Conservation in North America.

[102] Meyer S. (1991). Being Kind to Animal Pests. A Non-Nonsense Guide to Humane Animal Control with Cage Traps.

[103] Southworth P. (2018). Moment a Hunter Has to Free the Hissing and Clawing Mountain Lion He Trapped by Mistake While Trying to Catch Coyotes in the Utah Wilderness. Daily Mail.

[104] Alaska Outdoors Supersite Methods for dispatching trapped animals. http://forums.outdoorsdirectory.com/showthread.php/136907-Methods-for-Dispatching-Trapped-Animals.

[105] Wildlife removal, San Jose, CA. http://www.sanjosepestwildlife.com/squirrel-kill.html.

[106] Taxidwermy.net—Forum Dispatching Trapped Animals with Minimal Skull/Hide Damage. https://www.taxidermy.net/threads/356106/.

[107] Warburton B. (2015). Leghold Traps. A Guideline for Capturing Possums, Ferrets and Feral Cats Using Leghold Traps.

[108] American Association of Zoo Veterinarians (AAZV) (2006). Guidelines for Euthanasia of Nondomestic Animals.

[109] Leary S., Underwood W., Anthony R., Cartner S., Grandin T., Greenacre C., Gwaltney-Brant S., McCrackin M.A., Meyer R., Miller D. (2020). AVMA Guidelines for the Euthanasia of Animals.

[110] Vantassel S., Hiller T.L., Powell K.D.J., Hyngstrom S.E. (2010). Using advancements in cable-trapping to overcome barriers to furbearer management in the United States. J. Wildl. Manag..

[111] Larkin R.P., VanDeelen T.R., Sabick R.M., Gosselink T.E., Warner R.E. (2003). Electronic signaling for prompt removal of an animal from a trap. Wildl. Soc. Bull..

[112] Santos N., Rio-Maior H., Nakamura M., Roque S., Brandão R., Álvares F. (2017). Characterization and minimization of the stress response to trapping in free-ranging wolves (*Canis lupus*): Insights from physiology and behavior. Stress.

[113] Krause T. (1977). Editor’s notes. Am. Trapp..

[114] Miskosky R. (2016). Never let the truth get in the way of a good story. Alta. Outdoorsmen.

[115] Baker S.E., Macdonald D.W. (2012). Not so humane mole tube traps. Anim. Welf..

[116] The Animal Welfare Act 2006: What it means for wildlife. WML-GU02, England. https://assets.publishing.service.gov.uk/government/uploads/system/uploads/attachment_data/file/798010/wml-gu02-animal-welfare-act-wildlife-managment.pdf.

[117] Warburton B. (1982). Evaluation of seven trap models as humane and catch-efficient possum trap. N. Z. J. Zool..

[118] Krause T. (1989). Restraining trap research. Am. Trapp..

[119] Skinner D.L., Todd A.W. (1990). Evaluating efficiency of footholding devices for coyote capture. Wildl. Soc. Bull..

[120] Messineo J. (1991). The EGG (trap) and I. Am. Trapp..

[121] Hubert G.F., Hungerford L.L., Proulx G., Bluett R.D., Bowman L. (1996). Evaluation of two restraining traps to capture raccoons in non-drowning water sets. Wildl. Soc. Bull..

[122] Canadian General Standards Board (1996). Animal (Mammal) Traps—Mechanically Powered, Trigger-Activated Killing Traps for Use on Land.

[123] Proulx G. (1997). A preliminary evaluation of four types of traps to capture northern pocket gophers, *Thomomys talpoides*. Can. Field-Nat..

[124] Munõz-Igualada J., Shivik J.A., Domínguez F.G., González L.M., Aranda-Moreno A., Olalla M.F., García C.A. (2010). Traditional and new cable restraint systems to capture fox in central Spain. J. Wildl. Manag..

[125] Rodríguez A., Delibes M. (2004). Patterns and causes of non-natural mortality in the Iberian lynx during a 40-year period of range contraction. Biol. Conserv..

[126] Cabezas-Díaz S., Lozano J., Virgós E., Aronoff J.B. (2009). The declines of the wild rabbit (*Oryctolagus cuniculus*) and the Iberian lynx (*Lynx pardinus*) in Spain: Redirecting conservation efforts. Handbook of Nature Conservation: Global, Environmental and Economic Issues.

[127] Marshall C. (2015). Runners Injured in Animal Snares. BBC News Environment Correspondent. https://www.bbc.com/news/science-environment-32503789.

[128] Fur Institute of Canada (FIC) (1996). An Abridged Chronology of Events Concerning EU Regulation #3254/91.

[129] NAWAC guidelines 09 (2019). Assessing the Welfare Performance of RESTRAINING and kill Traps. https://www.mpi.govt.nz/dmsdocument/8521-nawac-guideline-09-assessing-the-welfare-performance-of-restraining-and-kill-traps.

[130] Cook S.R., Proulx G. (1989). Use of a digital waveform analyzer, accelerometers, and a load cell to measure momentum and clamping forces of killing traps for furbearers. Astm. J. Test. Eval..

[131] Cattet MRL (2013). Falling through the cracks: Shortcomings in the collaboration between biologists and veterinarians and their consequences for wildlife. ILAR J..

[132] McDonald J. (2012). Cornerstone of U.S. conservation: The Pitman-Robertson Act celebrates 75 years. Wildl. Prof..

[133] Dronova N., Shestakov A.S. (2015). Conservation and Socioeconomic Aspects of the Fur Trade in the Russian Far East. TRAFFIC Europe–Russia. Rufford Maurice Laing Foundation and WWF Germany. ttps://www.researchgate.net/publication/305281177_Trapping_a_Living_Conservation_and_Socio-Economic_Aspects_of_the_Fur_Trade_in_the_Russian_Far_East.

